# Artificial intelligence can accurately distinguish IgA nephropathy from diabetic nephropathy under Masson staining and becomes an important assistant for renal pathologists

**DOI:** 10.3389/fmed.2023.1066125

**Published:** 2023-07-03

**Authors:** Zhenliang Fan, Qiaorui Yang, Hong Xia, Peipei Zhang, Ke Sun, Mengfan Yang, Riping Yin, Dongxue Zhao, Hongzhen Ma, Yiwei Shen, Junfen Fan

**Affiliations:** ^1^Nephrology Department, The First Affiliated Hospital of Zhejiang Chinese Medical University (Zhejiang Provincial Hospital of Traditional Chinese Medicine), Hangzhou, China; ^2^Academy of Chinese Medical Science, Zhejiang Chinese Medical University, Hangzhou, China; ^3^Harbin Institute of Physical Education, Harbin, China; ^4^Graduate School, Zhejiang Chinese Medical University, Hangzhou, China; ^5^Chengdu University of Traditional Chinese Medicine, Chengdu, China; ^6^Nephrology and Endocrinology Department, Pinghu Hospital of Traditional Chinese Medicine, Pinghu, China; ^7^Ningbo Municipal Hospital of Traditional Chinese Medicine (Affiliated Hospital of Zhejiang Chinese Medical University), Ningbo, China

**Keywords:** artificial intelligence, IgA nephropathy, diabetic nephropathy, Yolov5 6.1, renal pathology

## Abstract

**Introduction:**

Hyperplasia of the mesangial area is common in IgA nephropathy (IgAN) and diabetic nephropathy (DN), and it is often difficult to distinguish them by light microscopy alone, especially in the absence of clinical data. At present, artificial intelligence (AI) is widely used in pathological diagnosis, but mainly in tumor pathology. The application of AI in renal pathological is still in its infancy.

**Methods:**

Patients diagnosed as IgAN or DN by renal biopsy in First Affiliated Hospital of Zhejiang Chinese Medicine University from September 1, 2020 to April 30, 2022 were selected as the training set, and patients who diagnosed from May 1, 2022 to June 30, 2022 were selected as the test set. We focused on the glomerulus and captured the field of the glomerulus in Masson staining WSI at 200x magnification, all in 1,000 × 1,000 pixels JPEG format. We augmented the data from training set through minor affine transformation, and then randomly split the training set into training and adjustment data according to 8:2. The training data and the Yolov5 6.1 algorithm were used to train the AI model with constant adjustment of parameters according to the adjusted data. Finally, we obtained the optimal model, tested this model with test set and compared it with renal pathologists.

**Results:**

AI can accurately detect the glomeruli. The overall accuracy of AI glomerulus detection was 98.67% and the omission rate was only 1.30%. No Intact glomerulus was missed. The overall accuracy of AI reached 73.24%, among which the accuracy of IgAN reached 77.27% and DN reached 69.59%. The AUC of IgAN was 0.733 and that of DN was 0.627. In addition, compared with renal pathologists, AI can distinguish IgAN from DN more quickly and accurately, and has higher consistency.

**Discussion:**

We constructed an AI model based on Masson staining images of renal tissue to distinguish IgAN from DN. This model has also been successfully deployed in the work of renal pathologists to assist them in their daily diagnosis and teaching work.

## 1. Introduction

IgA nephropathy (IgAN) and diabetic nephropathy are also receiving increasing attention as the most common primary and secondary causes of CKD (1–3). IgAN is a glomerulonephritis with microscopic hematuria and IgA deposition in the glomerular as the main clinical and pathological manifestations. Diabetic nephropathy is a microvascular complication of diabetes mellitus, with proteinuria and renal function decline as the main manifestations. Although most diabetic nephropathy can be accurately diagnosed based on the history and typical manifestations, renal pathology is still the gold standard for the diagnosis of diabetic nephropathy (4). In the early stage of diabetic nephropathy, the glomerulus mainly presents with thickening GBM and hyperplasia of mesangial area, while in the middle-late stage, the formation of nodular sclerosis and even glomerulosclerosis can be seen.

Although IgA nephropathy and diabetic nephropathy are primary and secondary glomerular diseases respectively, they both have prominent mesangial hyperplasia in pathological manifestations. The histological features of IgA nephropathy show significant individual variation in light microscopy, but most are characterized by the proliferation of mesangial cells and mesangial matrix, and fuchsinophilic deposition in Masson staining. The histological features of diabetic nephropathy in light microscopy are diffuse thickening GBM, hyperplasia of mesangial area, nodular sclerosis (K-W nodules) and glomerulosclerosis, as well as interstitial fibrosis, renal tubule atrophy and arteriolar hyaline. However, many patients with diabetes do not have typical clinical symptoms, and even do not know whether they have diabetes when undergoing kidney biopsy. Lack of clinical diagnosis of diabetes will undoubtedly lead to the neglect of diabetic nephropathy by pathologists in renal pathological diagnosis. Mesangial hyperplasia is common in both IgA nephropathy and diabetic nephropathy, and it is often difficult to distinguish them by light microscopy alone, especially in the absence of clinical data. Similarly, it is difficult to detect DN when patients have IgAN and early diabetic nephropathy.

Artificial intelligence (AI) is a branch of computer science, which mainly includes robotics, language recognition, image recognition, natural language processing and expert systems. In the last decade, computer vision technology has played an important role in the diagnosis of tumors. It has greatly helped pathologists and radiologists to diagnose cancer earlier and improve their efficiency (5–7). However, the application of AI in renal pathology is still in its infancy. Although many deep learning models have achieved high accuracy, they have not been applied into practical work (8–10). Because AI may be able to pick up diagnostic details missed by pathologists, we think it may play a role in distinguishing IgAN from diabetic nephropathy.

In this study, we constructed a computer vision model with Yolov5s based on renal pathology. Then, we used it to distinguish IgAN from DN and compared the result with renal pathologists. We found that AI can accurately differentiate IgAN from DN and assist pathologist to complete pathological diagnosis with higher diagnostic efficiency. Subsequently, we put this AI model into practical application to assist renal pathologists in their diagnosis and teaching. We believe that this is an innovative attempt of AI in kidney pathology, which will further help us explore the value of AI in kidney pathology.

## 2. Materials and methods

### 2.1. Patient information

This study was a retrospective study. Renal biopsy pathological sections of all patients were obtained from the First Affiliated Hospital of Zhejiang Chinese Medicine University. We selected patients diagnosed with IgAN or DN who underwent renal biopsy in our center from September 1, 2020 to April 30, 2022. We extracted whole slide imaging (WSI) of Masson staining of their renal pathology from the database as training set to build computer vision model. Patients who underwent renal biopsy from May 1, 2022 to June 30, 2022 and were diagnosed as IgAN or DN were selected as the test set. Subjects were included as follows: (1) the pathological diagnosis was primary IgA nephropathy or diabetic nephropathy; (2) the quality of Masson staining sections of the patient met the requirements of pathological diagnosis; (3) age 18 or older. Exclusion criteria included the following: (1) familial IgA nephropathy or IgA nephropathy due to other genetic factors; (2) IgA nephropathy combining with other glomerulonephritis or diabetic nephropathy; (3) diabetic nephropathy combining with other glomerular diseases; (4) Masson staining sections made from frozen tissue.

### 2.2. Masson staining section preparation and pathological diagnosis

Masson staining section preparation: The kidney tissue fixed with formalin was dehydrated, embedded in paraffin and cut into 3 μm sections. Staining was performed with hematoxylin solution and Masson cyaniding solution after rinsing. The slices were rinsed again, and then stained with red staining solution, washed with weak acid, dyed with aniline blue staining solution, and finally sealed with xylene transparent and neutral gum. Sections were scanned using a Leica digital Pathology slide scanner (Aperio GT 450).

Pathological diagnosis: The initial diagnosis of the patient’s renal pathology was made by the primary renal pathologist and the final diagnosis was determined by 2 senior renal pathologists. The diagnosis of IgA nephropathy and diabetic nephropathy is mainly based on light microscopy, immunofluorescence and electron microscopy. Under light microscope, mesangial cell hyperplasia accompanied by matrix increase was the basic lesion of IgAN, and immunofluorescence showed that anti-IgA antibody labeled immunofluorescence positive in the glomerular mesangial area showed clumps or coarse particles deposition. Under electron microscope, IgA nephropathy can be seen in the mesangial area and the parmesangial area of electron dense deposition lesions. The pathological diagnosis of diabetic nephropathy is the diffuse thickening of glomerular basement membrane, glomerular mesangial expansion, K-W nodules, glomerular exudative lesions, and arteriolar hyalinization under light microscope in patients with abnormal glucose metabolism. Immunofluorescence showed that IgG and albumin were thready positive along the glomerular basement membrane, Bowman’s capsule and some renal tubular basement membrane. Under electron microscopy, the characteristic lesions were homogeneous thickening of GBM and fusion of podocytic. All primary renal pathologist and senior pathologists will follow the above criteria for pathological diagnosis of patients.

### 2.3. Image preprocessing and image annotation

The quality of WSI in all patients was manually confirmed by a renal clinicopathologist. He will make sure there are no bubbles, folding compression, tearing, obvious knife chatter, staining deviations, and blurring in the WSI. The resolution of a WSI was as high as 32,768 × 32,768 pixels, so we chose manual interception to segment the WSI into small images. We focused on the glomerulus and captured the field of the glomerulus in WSI at 200× magnification, all in 1,000 × 1,000 pixels JPEG format. Finally, 2048 qualified IgA nephropathy and 1,071 diabetic nephropathy glomerular images were obtained in the training set, and120 qualified IgA nephropathy and 116 diabetic nephropathy glomerular images in the test set.

We used minor flip and rotation affine transformations as a data augmentation technique for the training set, and all the images of the training set will be rotated 90° and −90°, flipped horizontally and vertically. Finally, the training set consisted of 15,595 images.

All images were annotated manually by the renal pathologist using *Labelme.exe*. Annotation was completed by a primary pathologist and verified by a senior pathologist.

### 2.4. Framework and building methods of models

**Figure 1:**
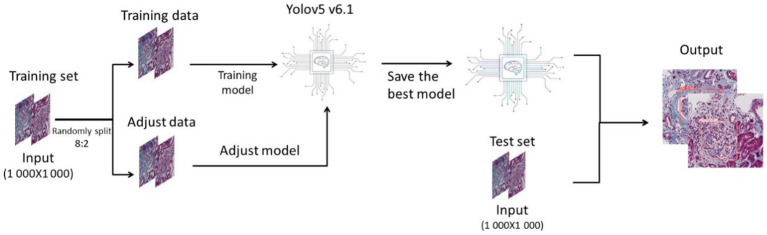
Flowcharts for deep learning and study plan.

Python3 is used as the programming language, and Yolov5 V6.1 is used as the deep learning framework. In this study, we randomly divided the pictures of the training set into training data and adjustment data in a ratio of 8:2. We used the training data to build the model, and used the adjustment data to continuously test the model and adjust the parameters and structure until the optimal deep learning model was output. Then we used the test set to test the deep learning model and made a final evaluation of the model (Figure 1).

The batch size for training is 16 and the eopchs for training are 300. More detailed hyperparameter Settings can be found in the Supplementary material 1.

### 2.5. Diagnostic comparison

To compare our model’s ability to distinguish between IgAN and diabetic nephropathy with renal pathologists, we invited different grades of renal pathologists to complete the test. We randomly selected 25 IgAN and 25 diabetic nephropathy images from the test set to form our test data. These test images were sent online to different grades of renal pathologists at three well-known renal clinical centers in China.^1^ We compared the results of AI and pathologist judgments to evaluate the value of our model in actual renal pathology work.

### 2.6. Statistical analysis

Statistical analysis was performed by IBM-SPSS25. Categorical data were represented by *N* (*n* %), and continuous variables were represented by *P*_50_(*P*_25_–*P*_75_). The Fisher exact or Wlicoxon rank sum test was used for hypothesis testing between training and test set patients. ROC curve is currently the most used analytical method to test the accuracy of diagnostic tools, and the curve closer to the top left indicates that the model is more accurate. AUC is the area under the ROC curve, which is a performance index to measure the quality of the model. The Youden index is a method for evaluating the authenticity of screening tests. It represents the total ability of the model to detect patients with true IgA nephropathy or diabetic nephropathy. The purpose of Kappa consistency test is to compare whether the results obtained by different methods are consistent, and it is also the most used method to judge consistency in the field of AI. Therefore, in this study we use ROC curve, AUC area and Youden index were used to evaluate the diagnostic efficacy of the deep learning model, and Kappa consistency test was used to evaluate the consistency of the AI with renal pathologists, *p* < 0.05 was considered statistically significant.

## 3. Result

### 3.1. AI can accurately detect the glomerulus

In our study, 167 patients with primary IgA nephropathy and 104 patients with diabetic nephropathy were included as the training set, and 19 patients with IgA nephropathy and 9 patients with diabetic nephropathy were included as the test set. No statistically significant differences were observed between the training and test set patients for either IgA or diabetic nephropathy (Tables 1, 2).

**Table 1:** Clinical information of patients with IgA nephropathy.

	Training set (167)	Test set (19)	Total (186)	*p*-value
Male (%)	82 (49%)	13 (68%)	95 (51%)	0.221
Hypertension (%)	70 (42%)	10 (53%)	80 (43%)	0.465
Diabetes (%)^*^	14 (8%)	4 (21%)	18 (10%)	0.226
Hepatitis B (%)	16 (10%)	2 (11%)	18 (10%)	0.987
Age	41 (32–53)	36 (32.0–47)	40 (32–52)	0.489
Height (cm)	165 (160–171)	168 (162.5–174)	165 (160–171.8)	0.063
Weight (cm)	63.0 (56.0–74.3)	68.9 (58.4–79.5)	64.9 (56.0–74.6)	0.223
BMI	23.3 (21.0–26.3)	24.5 (21.2–27.0)	23.7 (21.0–26.4)	0.502
Systolic blood pressure (mmHg)	129 (119–146)	142 (126–152)	130 (121–147)	0.069
Diastolic blood pressure (mmHg)	82 (73–90)	84 (73–96)	82 (73–91)	0.520
Heart rate	86 (78–93)	89 (78–98)	86 (78.0–94)	0.519
Fasting blood glucose (mmol/L)	4.8 (4.5–5.3)	4.7 (4.3–6.1)	4.8 (4.4–5.4)	0.939
Hemoglobin	130.0 (116.5–141.8)	130.0 (109.5–149.5)	130.0 (114.0–144.0)	0.825
High sensitivity C-reactive protein (mg/L)	1.1 (1–2.6)	1.0 (1.0–1.8)	1.0 (1.0–2.4)	0.310
Uric acid (umol/L)	358 (292–441)	395 (345–436)	370.5 (298–440)	0.544
Creatinine (umol/L)	83 (63–116)	95 (74–155)	87 (67–123)	0.125
Urea nitrogen (mmol/L)	5.2 (4.5–7.2)	6.3 (4.6–9.0)	5.4 (4.5–8.1)	0.156
Albumin (g/L)	37.8 (34.1–41.8)	37.1 (33.7–42.7)	37.5 (34.0–42.0)	0.768
Cholesterol (mmol/L)	4.9 (3.9–5.6)	4.8 (4–6.1)	4.9 (3.9–5.6)	0.656
Triglycerides (mmol/L)	1.5 (1.0–2.1)	1.4 (1.0–1.9)	1.5 (1.0–2.1)	0.585
Hemoglobin A1c (%)	5.6 (5.4–6.0)	5.7 (5.5–5.9)	5.6 (5.4–6.0)	0.658
Urine red blood cell (/uL)	116.8 (27.8–297.0)	55.6 (22.3–133.5)	107.3 (27.8–246.0)	0.156
24 h urinary protein (mg)	647.1 (254.8–2102.9)	1183.1 (351.2–3382.4)	703.8 (262.7–2558.7)	0.317
Urinary albumin/creatinine (mg/mgCr)	0.4 (0.1–1.2)	0.2 (0.1–1.5)	0.4 (0.1–1.2)	0.655

^*^Definite diagnosis of diabetes before the kidney biopsy is performed.

**Table 2:** Clinical information of patients with diabetic nephropathy.

	Training set (104)	Test set (9)	Total (113)	*p*-value
Male (%)	78 (75%)	8 (89%)	86 (76%)	0.370
Hypertension (%)	83 (80%)	8 (89%)	91 (81%)	0.626
Diabetes (%)^*^	78 (75%)	7 (78%)	85 (75%)	0.683
Hepatitis B (%)	10 (10%)	0 (0%)	10 (9%)	0.527
Age	58 (52.3–67)	59 (46.5–64.5)	58 (51–66.5)	0.803
Height (cm)	165 (163–172)	168.5 (165.8–171.5)	168 (163.3–171.5)	0.477
Weight (cm)	71.9 (60.2–81.8)	70.2 (68.0–78.2)	71.3 (60.8–80.5)	0.863
BMI	24.7 (22.6–27.9)	24.9 (23.2–27.0)	24.8 (22.6–27.3)	>0.999
Systolic blood pressure (mmHg)	141.5 (126.3–157.0)	137.5 (113.3–152.0)	141.5 (122.3–156.3)	0.477
Diastolic blood pressure (mmHg)	79.5 (72.0–87.0)	82 (70.8–89.0)	80.5 (72.0–87.0)	0.833
Heart rate	80.5 (69.3–88.8)	78 (67–95)	79 (69.3–92.3)	0.687
Fasting blood glucose (mmol/L)	5.5 (4.2–7.7)	4.8 (4.5–5.3)	5.6 (4.5–7.5)	0.716
Hemoglobin	118 (99–141.5)	130 (116.5–141.8)	121.5 (97.3–144.5)	0.659
High sensitivity C-reactive protein (mg/L)	1.6 (1–5.5)	1.1 (1–2.6)	1.6 (1–4.4)	0.803
Uric acid (umol/L)	368 (342.3–486.5)	358 (292–441)	405.5 (357.8–487.5)	0.272
Creatinine (umol/L)	117.5 (81.5–187.8)	83 (63–116)	139 (95.5–244.8)	0.091
Urea nitrogen (mmol/L)	8.8 (5.8–11.8)	5.2 (4.5–7.2)	8.2 (5.8–11.9)	0.346
Albumin (g/L)	34.8 (28.2–37.6)	37.8 (34.1–41.8)	35.8 (28.8–41.4)	0.239
Cholesterol (mmol/L)	4.1 (3.1–5.2)	4.9 (3.9–5.6)	4.1 (3.2–5.2)	0.889
Triglycerides (mmol/L)	2 (1.2–3.1)	1.5 (1–2.1)	1.7 (1.2–2.6)	0.367
Hemoglobin A1c (%)	6.5 (5.8–8.4)	5.6 (5.4–6)	6.7 (6.1–7.9)	0.616
Urine red blood cell (/uL)	13.8 (5.6–33.4)	116.8 (27.8–297)	13.8 (5.6–33.4)	0.604
24 h urinary protein (mg)	3047.3 (271.1–4738.8)	647.1 (254.8–2102.9)	2306.5 (225.5–4681.6)	0.415
Urinary albumin/creatinine (mg/mgCr)	2.6 (0.1–4.4)	0.4 (0.1–1.2)	1.2 (0.1–3.7)	0.307

^*^Definite diagnosis of diabetes before the kidney biopsy is performed.

### 3.2. AI can accurately detect the glomerulus

In the test set, the 120 IgAN images contained a total of 184 glomerulus (including incomplete glomerulus at image margins). A total of 177 glomerulus were detected by AI. All intact glomerulus in the image were successfully detected, except for 1 renal tubule which was misjudged as glomeruli and 8 incomplete glomerulus at the edge of the image. One hundred and sixteen images of diabetic nephropathy contained 200 glomeruli (including incomplete glomerulus at image margins). A total of 198 glomerulus were detected by AI. In the image, all intact glomeruli were successfully detected, and 4 were misdiagnosed as glomerulus by tubules or renal inerestitum. The AI missed 6 glomerulus, including 4 of which were completely sclerosed and atrophic, and 2 were incomplete glomerulus at the edge of the image. The overall accuracy of AI glomerulus detection was 98.67%, and the omission rate was only 1.30% without the omittance of intact glomeruli (Table 3).

**Table 3:** AI locates glomerular conditions.

	The actual number of glomeruli	Number of glomeruli detected	Misjudged	Missed
IgA nephropathy	184	177	1	8
Diabetic nephropathy	200	198	4	6
Total	384	375	5	14

### 3.3. AI can distinguish IgAN and diabetic nephropathy

We analyzed the accuracy of AI in differentiate IgAN from diabetic nephropathy, and the overall accuracy of AI reached 73.24%, among which the accuracy of IgAN reached 77.27%, and diabetic nephropathy reached 69.59%. The possibility of misjudged prediction of IgAN and diabetic nephropathy was 30.26 and 22.56%, respectively (Table 4).

**Table 4:** AI predicted glomerular classification accuracy.

		True glomerular classification		
		IgAN	Diabetic nephropathy	Total
AI judgment classification	IgAN	136	59	195
	Diabetic nephropathy	40	135	175
	Total	176	194	370

**Figure 2:**
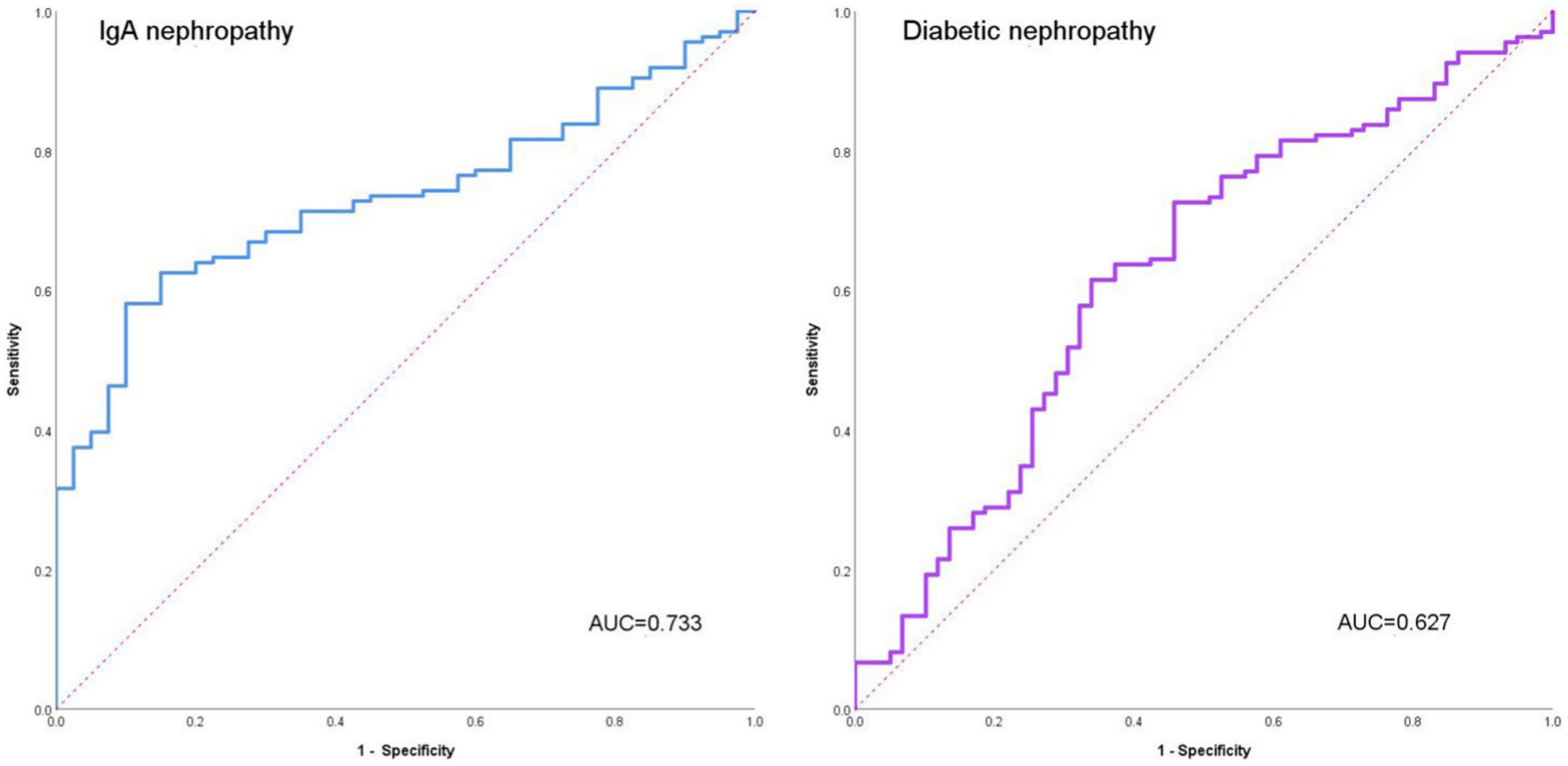
The ability of AI to distinguish IgAN from diabetic nephropathy.

We separately analyzed the ability of AI to distinguish IgAN from diabetic nephropathy (Figure 2). The accuracy of AI for IgA nephropathy was 0.732, the precision was 0.697, the recall was 0.773, and F1 score was 0.733. The accuracy of AI for diabetic nephropathy was 0.732, the precision was 0.771, the recall was 0.696, and F1 score was 0.732. The AUC of IgA nephropathy was 0.733 (95% CI 0.659–0.808, *p* < 0.001) and that of diabetic nephropathy was 0.627 (95% CI 0.541–0.714, *p* = 0.005). This indicates that AI can autonomously extract effective information from images and extract key features from limited information, thus accurately distinguishing IgAN from diabetic nephropathy.

### 3.4. Compare the ability of AI and renal pathologists to distinguish between IgAN and diabetic nephropathy

In this study, we invited 5 primary renal pathologists, 3 intermediate pathologists, and 2 senior pathologists to participate in our test. All the renal pathologists come from three famous renal clinical center in Harbin, Nanjing and Xi’an. They all took an online test. They were asked to distinguish between 25 IgAN and 25 diabetic nephropathy using Masson’s stain (×200). The time to complete the test and accuracy were analyzed and compared with the AI.

**Figure 3:**
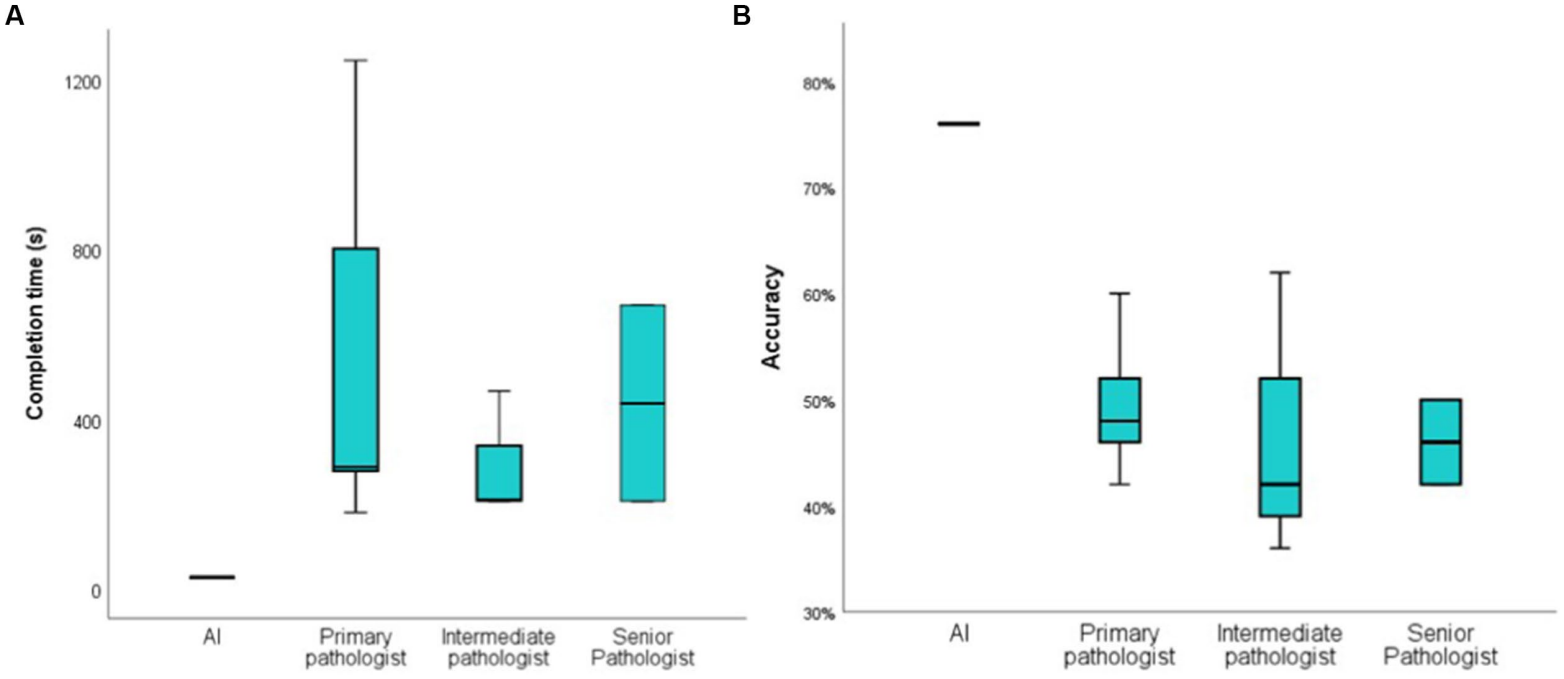
Compare the time and accuracy of diagnosis between AI and pathologist. **(A)**: the time it takes for the AI and pathologist to distinguish between 50 images; **(B)**: the accuracy of the AI and pathologist to distinguish between 50 pathological images.

It took pathologists about 3 to 14 min to complete the test, and even one primary pathologist spent more than 20 min, but AI only took 26 s overall, averaging 0.52 s per image (Figure 3A). Senior pathologists did not perform better than primary or intermediate pathologists in terms of accuracy. The accuracy rate was 49.6% ± 6.84% for primary pathologists, 46.67% ± 13.61% for intermediate pathologists and 46% ± 5.66% for senior pathologists. We also compared the accuracy between pathologists and AI in distinguishing IgAN from diabetic nephropathy (Figure 3B). However, there is significant randomness in the pathologist’s judgment, the AI was more consistent than the pathologists (Table 5), a specific comparison can be found in Supplementary material 2.

**Table 5:** Compare the ability of AI and renal pathologists.

	Completion time (s)	Accuracy	*κ*	Youden index
Primary pathologist 1	1,246	46.00%	−0.08	−0.08
Primary pathologist 2	179	42.00%	−0.16	−0.16
Primary pathologist 3	287	52.00%	0.04	0.04
Primary pathologist 4	278	60.00%	0.20	0.20
Primary pathologist 5	802	48.00%	−0.04	−0.04
Intermediate pathologists 1	209	36.00%	−0.28	−0.28
Intermediate pathologists 2	466	62.00%	0.24	0.24
Intermediate pathologists 3	206	42.00%	−0.16	−0.16
Senior pathologists 1	206	50.00%	0.01	0
Senior pathologists 2	668	42.00%	−0.16	−0.16
AI	26.36	76.00%	0.52	0.52

## 4. State of the art

“Artificial intelligence” appeared in the summer of 1956 after more than 60 years and it has become a nearly ubiquitous part of our day-to-day lives. In the past decade, AI has been increasingly used in automated diagnosis, hospital management, and wearable devices. However, the application of AI in pathological diagnosis is an emerging interdisciplinary discipline in the last decade, among which AI has been most widely and successfully applied in tumor pathological diagnosis. Current successful cases demonstrated that AI can extract effective information from megapixel images, and then pay more attention to morphological changes of sub-vision beyond human eyes and finally make correct judgments. The application of AI in renal pathology diagnosis is still under research.

Basso and his colleagues used AI to extract 233 kinds of interpretable biological information, such as color, morphology, microstructure and texture, from 45 patients. They used these biomarkers to construct a machine learning model, and used this model to successfully distinguish minimal change disease, membranous nephropathy and thin basement membrane nephropathy (11). Kers et al. (12) used convolutional neural networks to analyze 5,844 digital whole slide images of 1948 kidney transplant patients. Their research confirmed that deep learning algorithm such as convolutional neural networks also have great advantages in the pathology of kidney allograft biopsies, AI can assist renal pathologists to diagnose rejection as early as possible, and play a greater role with human-computer interaction in the future. Zeng et al. (8) tried to use artificial intelligence to assist in the identification and quantitative analysis of kidney lesions. They used deep convolutional neural networks and biomedical image processing algorithms to locate glomeruli in renal pathology images of 400 Chinese IgAN patients, distinguish and quantify different cells and further identify characteristic lesions such as spherical sclerosis, segmental glomerulosclerosis, crescent, and finally quantify the glomerular lesions. Their study successfully used AI to quantify the severity of glomerular lesions, and it enables the application of AI in the use of renal pathological diagnosis. In the AI application of diabetic nephropathy, Kitamura and colleagues collected six immunofluorescence photos of 885 patients, including IgG, IgA, IgM, C3, C1q and fibrin, and used a convolutional neural network to classify. Their study found that AI could accurately diagnose DN using only immunofluorescence (10). They believe that this is related to the ability of artificial intelligence to find feature that are difficult to see by the naked eye, which is the biggest advantage of AI in assisting pathologists to complete the diagnosis in the future.

However, most of the studies on the application of AI in renal pathology mainly utilize the developed algorithms to train models (13–15). They regard the diagnosis and rating of renal pathology as a process of image detection and recognition, and adopt computer vision in this process, in which the two key points are feature extraction and judgment. Although these studies on artificial intelligence and renal pathological diagnosis have achieved great success, and some models have been successfully put into clinical practice, there are still many challenges. First, many studies in renal pathology are often based on a certain type of staining, which is same as our study. However, renal pathological diagnosis is one of the most complex branches of clinical pathology. Renal pathological diagnosis is usually made by pathologists integrating optical microscopy, immunofluorescence microscopy, electron microscopy, clinical information and even genetic testing data. The information carried on one stain is too limited to identify a particular disease. Another important challenge of deep learning algorithm for renal pathology is image annotation. At present, the application of AI in renal pathology mainly adopts supervised learning method. Although this method is accurate and efficient, it needs many accurately labeled data to train the model. This annotation is a task that requires an experienced and specially trained pathologist. A robust model requires a lot of training data, which is a very labor-intensive task. Inadequate, inconsistent or inaccurate data annotation will seriously interfere with the accuracy of the model. For example, in our study we required that all images were annotated by a primary pathologist and reviewed by a senior pathologist to ensure that their labels were accurate. Last but not least, deep learning relies heavily on the development of algorithms and hardware updates. Deeper, more parametric networks and larger scale training data often yield more robust models, but this requires more advanced algorithms and hardware.

Chat Generative Pre-Trained Transformer (ChatGPT) is probably the hottest AI feature of the last 3 months, it is a natural language processing tool developed by OpenAI using the Transformer neural network architecture. The developers trained their models on a large corpus which include of real-world conversations, and finally giving ChatGPT the ability of language understanding and text generation. ChatGPT’s great performance does not just make it possible to converse freely with humans, but also can write emails and papers. However, ChatGPT is a model for processing sequential data, which is very different from the computer vision used in pathological diagnosis, and it does not have the advantage of processing matrix data such as pathological images. And the current research suggests that AI cannot really see, think, and solve problems like a scientist (16–18). We also believe that it is impossible for artificial intelligence to replace pathologists to independently complete the diagnosis of renal pathology. However, due to the superior performance of AI, we also believe that artificial intelligence will have a breakthrough development in renal pathological diagnosis and become the best assistant of renal pathologists in the future.

## 5. Discussion

At present, diabetic nephropathy is one of the most common causes of CKD, accounting for 30%–50% of CKD patients worldwide (19). The incidence of IgA nephropathy is also much higher than that of other primary glomerulonephritis. However, both IgAN and diabetic nephropathy are often characterized by mesangial hyperplasia, which makes it difficult to distinguish IgAN from diabetic nephropathy in practice, especially when clinicians are unaware of the patient’s history of diabetes.

As a new technology, AI has attracted more and more attention, especially in the field of pathological diagnosis. In the field of kidney pathology, pathologists and AI engineers have also made some attempts and achieved research results (10, 12, 20–23). However, many studies have stayed on the earliest convolutional neural network algorithm structure, which has great limitations in terms of operation speed and learning depth. Yolov5 V6.1 network framework is one of the most rapidly developing algorithmic frameworks in computer vision. In many computer vision fields, Yolov5 V6.1 shows advantages that other algorithms are difficult to compare. But there is still no example of the application of this algorithm structure in kidney pathology. Therefore, we try to use Yolov5 V6.1 network framework to deploy our algorithm in this study.

**Figure 4:**
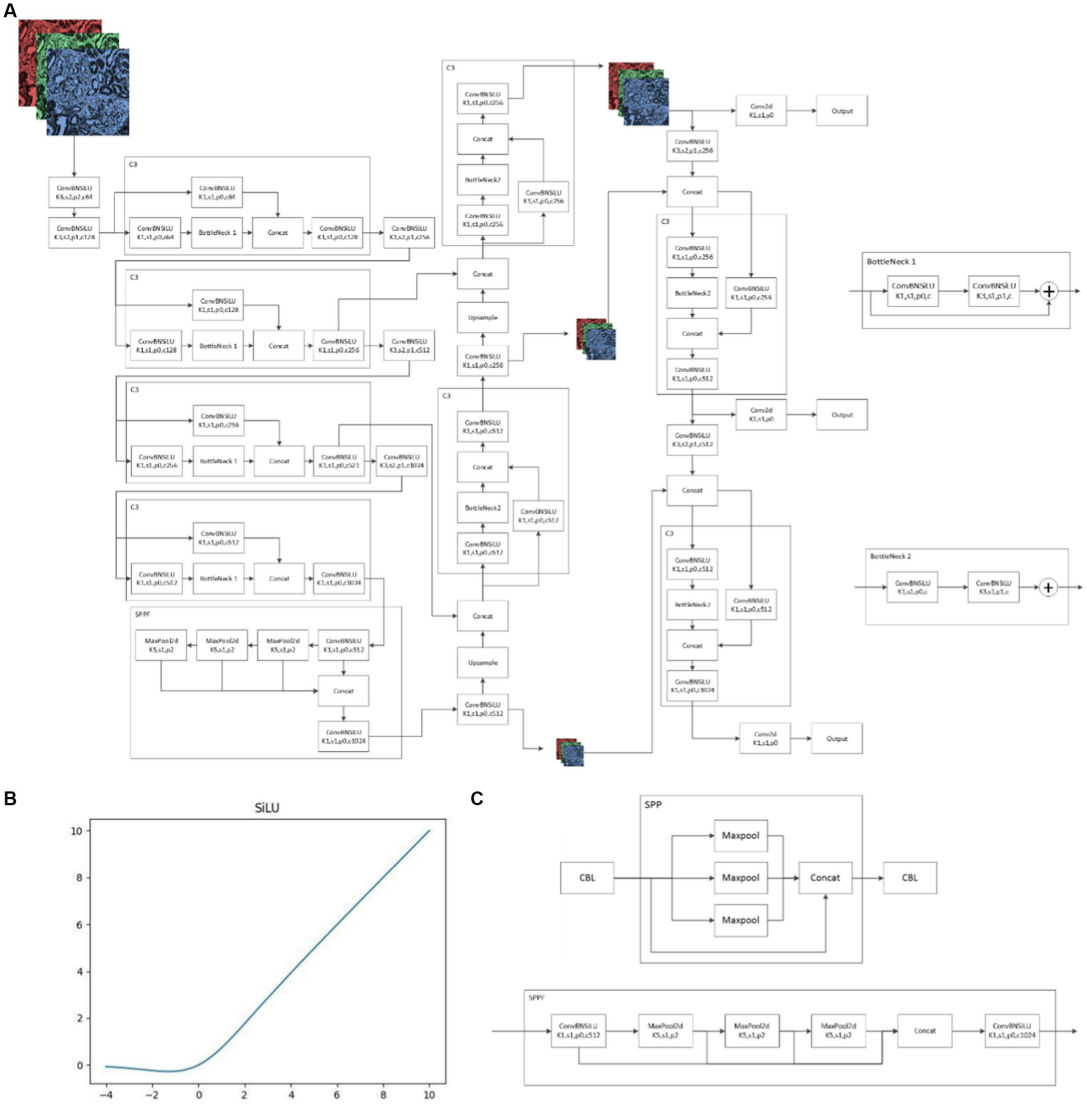
Yolov5 6.1 Network structure **(A)**: schematic diagram of Yolov5 6.1 network structure; **(B)**: SiLU function; **(C)**: differences between SPP and SPPF.

Yolov5 V6.1 Network structure consists of backbone, neck and head (Figure 4A). Backbone is mainly used to extract features, while neck mainly plays a role of connection. Neck mixes and combines the information extracted by backbone and transmits the information to the prediction layer, and head finally makes predictions. Yolov5 V6.1 structure is greatly optimized compared with other algorithm structures or earlier versions of Yolo. First, Yolov5 V6.1 deleted the original focus layer, but replaced it with a convolution layer with kernel = 6, stride = 2 and padding = 2. This operation provided great convenience for the deployment of algorithms and greatly improved the speed of our model. In addition, the activation functions adopted by Yolov5 V6.1 were SiLU (Figure 4B). SiLU function is characterized by no upper bound, but lower bound, smooth and non-monotonic, which makes the convergence speed of our model much faster than other models and more suitable for learning deeper network. Last, the SPP structure was replaced by the SPPF structure in Yolov5 V6.1 (Figure 4C). SPP structure is a key part of Yolo. It can change feature images of arbitrary size into feature vectors of fixed size, which is helpful to solve the problem of large difference of target sizes in detection images. However, SPPF can improve the running speed with the same effect of SPP, especially suitable for complex multi-target detection. The combination of SiLU and SPPF ensures the accuracy and efficiency of the model and enables the model to accurately distinguish glomerular diseases manifested as mesangial hyperplasia. Specific information on the structure of the algorithm is in Supplementary material 3.

**Figure 5:**
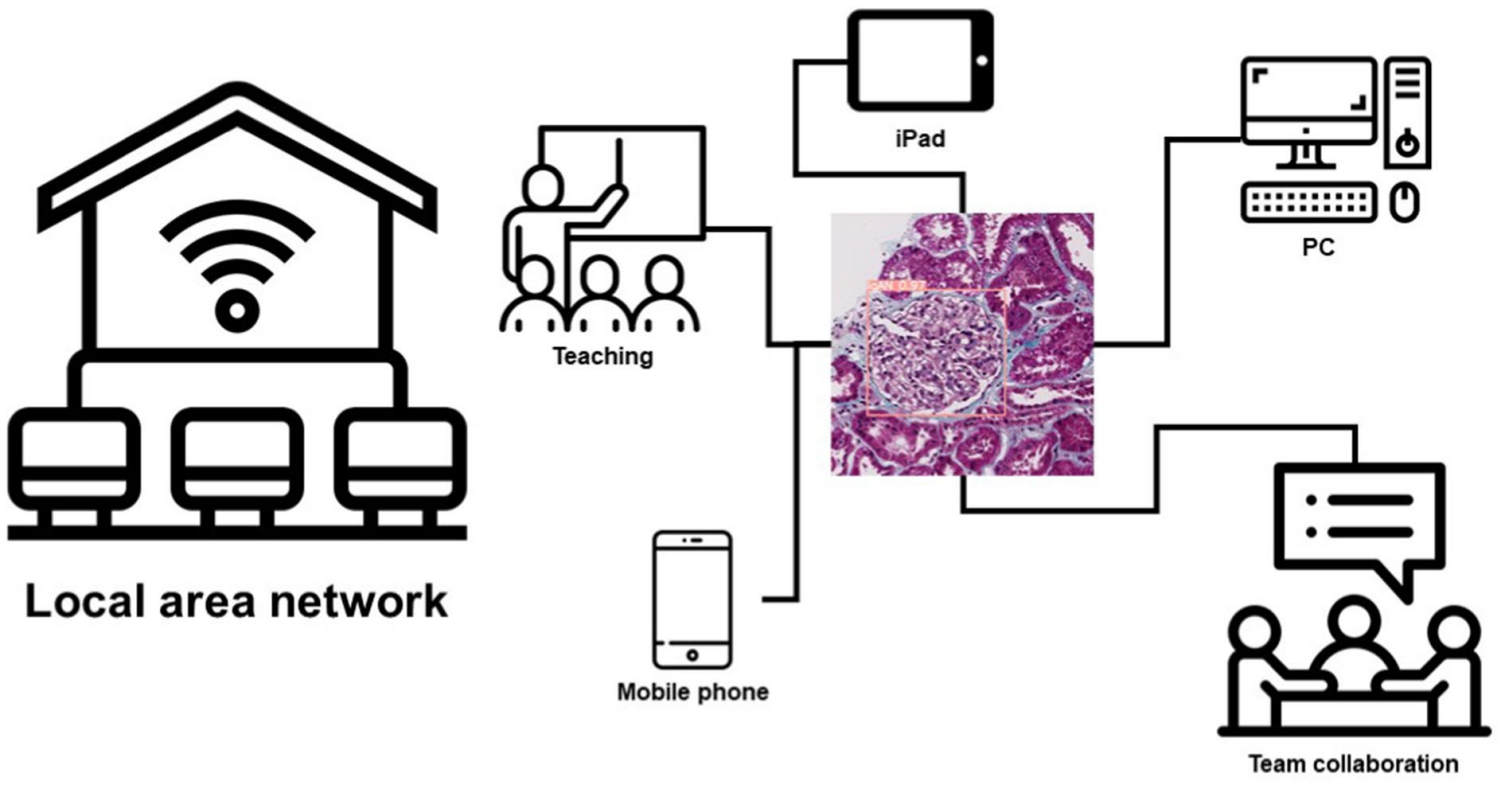
Schematic diagram of the model deployed in the LAN.

Another important aspect of AI is the deployment of models. Because of this, we tried a lot to improve the usability of the model. We use cloud computing technology to deploy our model on a local area network. Any PC, smartphone, or iPad connected to the local area network can use this model to make predictions. This brings great convenience to the teamwork and teaching of renal pathologists in their daily work, and this also provides a diagnostic aid for pathologists (Figure 5; Supplementary material 4).

Although, we also found that AI had higher consistent than renal pathologists, which we thought may be related to that AI can find some features in images that are difficult to recognize by the naked eye. We still believe that AI is only a tool to assist renal pathologists to complete their work, and they are far from being a substitute for renal pathologists to complete pathological diagnosis. As we have said before, renal pathological diagnosis cannot rely solely on a certain class of image features. It is not only the summary of light microscopy, immunofluorescence, electron microscopy and other image information, but also closely related to the clinical information of patients. Because of this, human expertise and the experience of clinical pathologists have an incomparable advantage to AI and play a decisive role in the final pathological diagnosis. Although AI facilitates pathologists’ work, we still need to be wary of the consequences of over-reliance on AI, which may interfere with pathologists’ thinking and increase the possibility of misdiagnosis of some atypical cases.

There are some limitations to our study. Firstly, only Masson staining was used in our study, and the image of other staining was not reported. As we mentioned earlier, the utilization of information has always been a key problem for computer vision in renal pathology, and how to stack up the information from multiple stains has been a problem for AI scientists. So that, we finally selected Masson staining as our study object, because Masson staining can reveal immune complex, extracellular matrix deposition and cell proliferation, which can be used to differentiate IgAN from DN. Secondly, we focused on the glomerulus to distinguish IgAN from DN, the information in tubulointerstitial lesions of IgAN and DN are ignore. As the pathological structure with the most prominent lesions in IgAN and DN, glomeruli is the area with the most abundant pathological information. In order to ensure a good generalization effect of our model, we only labeled glomeruli while proactively neglected the tubulointerstitial lesions to prevent overfitting. Third, we did not use clinical data of patients in the model, which may be useful for clinicians. Some physical examination or laboratory tests can also provide information for the diagnosis of IgA nephropathy and diabetic nephropathy. For example, the presence of diabetic retinopathy on funduscopic examination suggests that the patient may have diabetic nephropathy, while the presence of a history of chronic mucosal infectious diseases and episodic hematuria may be the manifestation of IgA nephropathy. However, we believe that this information, like our AI model, can only partially inform our diagnosis. The final diagnosis can only be the result of the combination of various aspects of information obtained by the renal pathologist and the clinician. In order to make our model accurately predict these patients, we did not add clinical data of patients in the model construction. We also believe that our AI model has superior performance and can well help renal pathologists to complete their diagnosis and improve their work efficiency. Fourth, although we tried many efforts to reduce the interference of potential confounders on model accuracy, we still could not eliminate all bias. We think that the most likely source of bias caused by subjective factors is the process of glomerular image extraction and annotation. Images extraction and annotation is a very boring and huge work, and the subjective deviation in this process may affect the final effect of the model. Nevertheless, many measures have been taken to reduce the interference of subjective factors in the process. For example, we required that all images be annotation by the same clinical pathologist who had been trained specifically for this study, ensuring that the glomerular interception and annotation processes were consistent. We also limited his working time every day and strictly ensured his rest to prevent bias caused by fatigue. From the perspective of our training process, the bias caused by the image annotation is acceptable.

## 6. Conclusion

In this study, we constructed an AI model based on Masson images of renal pathology that could be used to distinguish IgA nephropathy from diabetic nephropathy. Subsequently, we applied this model to renal pathologists’ daily work, playing an auxiliary role in diagnosis and teaching. However, there are still many challenges to be solved before AI can be used in renal pathology as widely and irreplaceably as it has been in tumor pathology. We hope this day will come soon as the algorithm structure is optimized and the hardware is updated.

## Data availability statement

The raw data supporting the conclusions of this article will be made available by the authors, without undue reservation.

## Ethics statement

The studies involving human participants were reviewed and approved by Ethics Committee of the First Affiliated Hospital of Zhejiang Chinese Medicine University. Written informed consent for participation was not required for this study in accordance with the national legislation and the institutional requirements.

## Author contributions

ZF participated in the design of the study, data collection and analysis, and the writing of the draft of the paper. QY, HX, PZ, and KS participated in the data collection. MY, RY, DZ, and YS participated in the data analysis. QY, JF, HM, and HX participated in constructing the model, writing the draft, and revising. All authors contributed to the article and approved the submitted version.

## Funding

This project is supported by the National Science Foundation of China (no. 82104756) and Science Foundation of Zhejiang province (no. LQ22H270002).

## Conflict of interest

The authors declare that the research was conducted in the absence of any commercial or financial relationships that could be construed as a potential conflict of interest.

## Publisher’s note

All claims expressed in this article are solely those of the authors and do not necessarily represent those of their affiliated organizations, or those of the publisher, the editors and the reviewers. Any product that may be evaluated in this article, or claim that may be made by its manufacturer, is not guaranteed or endorsed by the publisher.

## Footnotes

^1^ https://www.wjx.cn/

## Supplementary material

SUPPLEMENTARY MATERIAL 1 Details of the hyperparameter settings and learning process of the AI model.

SUPPLEMENTARY MATERIAL 2 Detailed results of renal pathologists distinguishing IgA nephropathy from diabetic nephropathy.

SUPPLEMENTARY MATERIAL 3 Yolov5 6.1 Network structure.

SUPPLEMENTARY MATERIAL 4 An example of our AI model in a clinical deployment.
