# Supplementary material for: Artificial intelligence can accurately distinguish IgA nephropathy from diabetic nephropathy under Masson staining and becomes an important assistant for renal pathologists
Source: Front Med (Lausanne). 2023 Jul 3;10:1066125. doi: 10.3389/fmed.2023.1066125 (PMC10352102; doi:10.3389/fmed.2023.1066125)
Supplement: SUPPLEMENTARY MATERIAL 1 — Details of the hyperparameter settings and learning process of the AI model. [file Data_Sheet_1.DOCX]

Yolov5 V6.1 Network structure consists of backbone, neck and head (*Supplement Figure1A*). Backbone is mainly used to extract features, while neck mainly plays a role of connection. Neck mixes and combines the information extracted by backbone and transmits the information to the prediction layer, and head finally makes predictions. Yolov5 V6.1 structure is greatly optimized compared with other algorithm structures or earlier versions of Yolo. First, Yolov5 V6.1 deleted the original focus layer, but replaced it with a convolution layer with kernel=6, stride=2 and padding=2. This operation provided great convenience for the deployment of algorithms and greatly improved the speed of our model. In addition, the activation functions adopted by Yolov5 v6.1 were SiLU (*Supplement Figure1B*). SiLU function is characterized by no upper bound, but lower bound, smooth and non-monotonic, which makes the convergence speed of our model much faster than other models and more suitable for learning deeper network. Last, the SPP structure was replaced by the SPPF structure in Yolov5 V6.1 (*Supplement Figure1C*). SPP structure is a key part of Yolo. It can change feature images of arbitrary size into feature vectors of fixed size, which is helpful to solve the problem of large difference of target sizes in detection images. However, SPPF can improve the running speed with the same effect of SPP, especially suitable for complex multi-target detection.


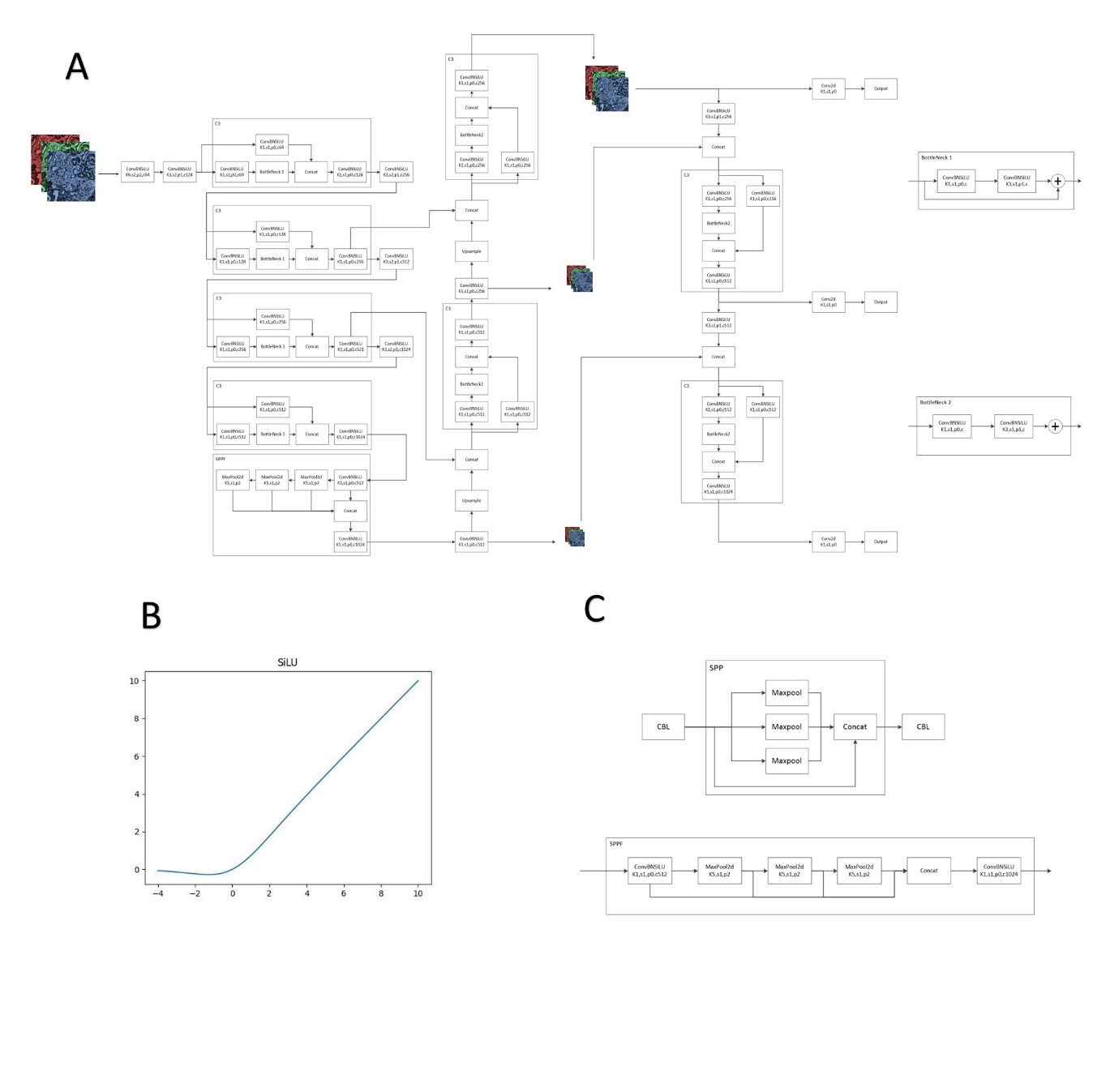


Supplement Figure 1. Yolov5 6.1 Network structure |A: Schematic diagram of Yolov5 6.1 network structure; B: SiLU function; C: Differences between SPP and SPPF

Yolov5 V6.1 has a more complex feature of extraction structure than the traditional convolutional neural network, which not only improves the feature extraction ability, but also improves the running speed. We also analyzed the advantage of YoloV5 V6.1 in distinguishing IgAN from diabetic nephropathy, and the use of SiLU function and SPPF structure may be an important reason for its excellent performance in this study. SiLU function has no upper boundary, which can avoid gradient saturation in deep learning. Meanwhile, it is smooth everywhere and allows some negative values, which makes the model maintain accuracy and generalization when learning depth is deepened. SPPF structure, like SPP, draws on the idea of spatial pyramid and solves the problem of image repetitive feature extraction while ensuring the running speed. The combination of SiLU and SPPF ensures the accuracy and efficiency of the model and enables the model to accurately distinguish glomerular diseases manifested as mesangial hyperplasia.
