# Supplementary material for: Artificial intelligence can accurately distinguish IgA nephropathy from diabetic nephropathy under Masson staining and becomes an important assistant for renal pathologists
Source: Front Med (Lausanne). 2023 Jul 3;10:1066125. doi: 10.3389/fmed.2023.1066125 (PMC10352102; doi:10.3389/fmed.2023.1066125)
Supplement: SUPPLEMENTARY MATERIAL 2 — Detailed results of renal pathologists distinguishing IgA nephropathy from diabetic nephropathy. [file Presentation_1.zip › Untitled Report _ YOLOv5 – Weights & Biases.pdf]

Share

Untitled Report

zyt

Section 1

Results

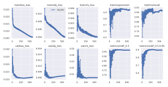
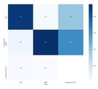
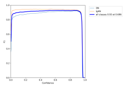
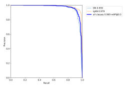
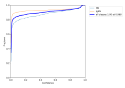
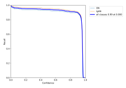

Validation

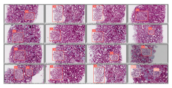
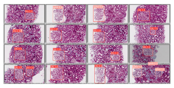
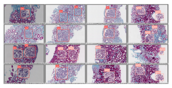
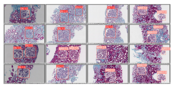
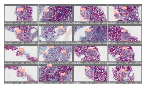
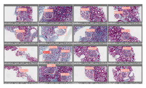

metrics/recall

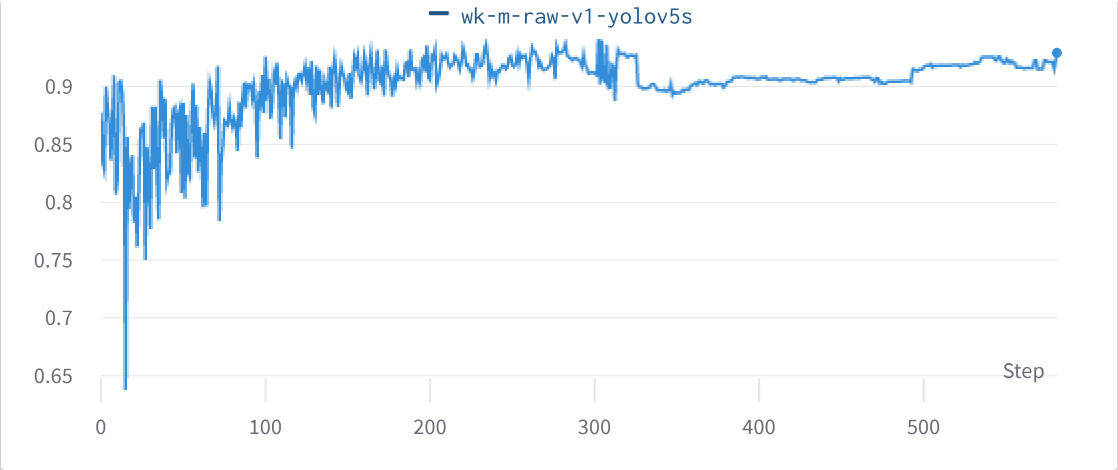
wk-m-raw-v1-yolov5s
0.9
0.85
0.8
0.75
0.7
0.65
Step
0
100
200
300
400
500

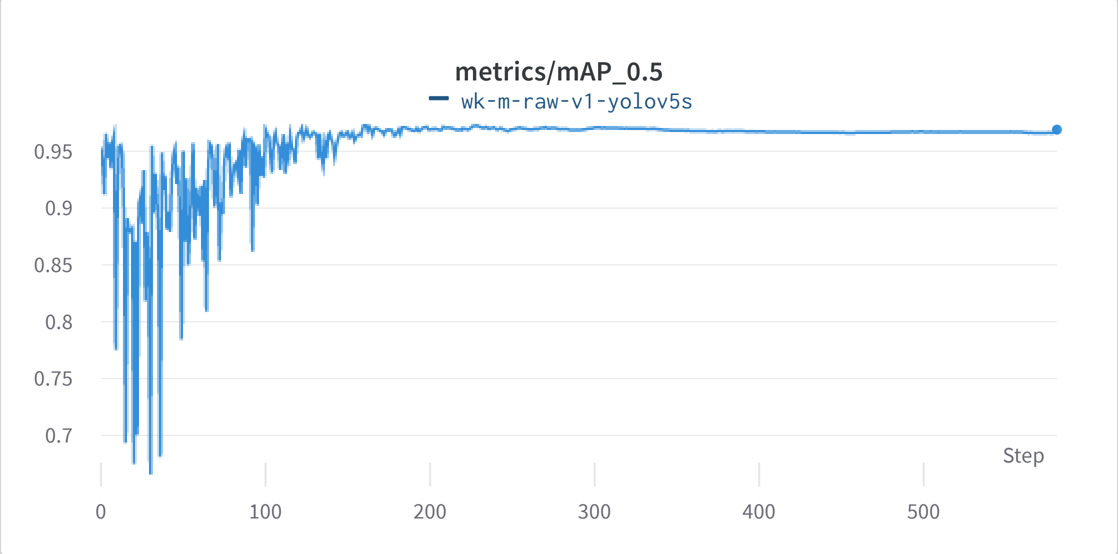
metrics/mAP_0.5
wk-m-raw-v1-yolov5s
0.95
0.9
0.85
0.8
0.75
0.7
Step
0
100
200
300
400
500

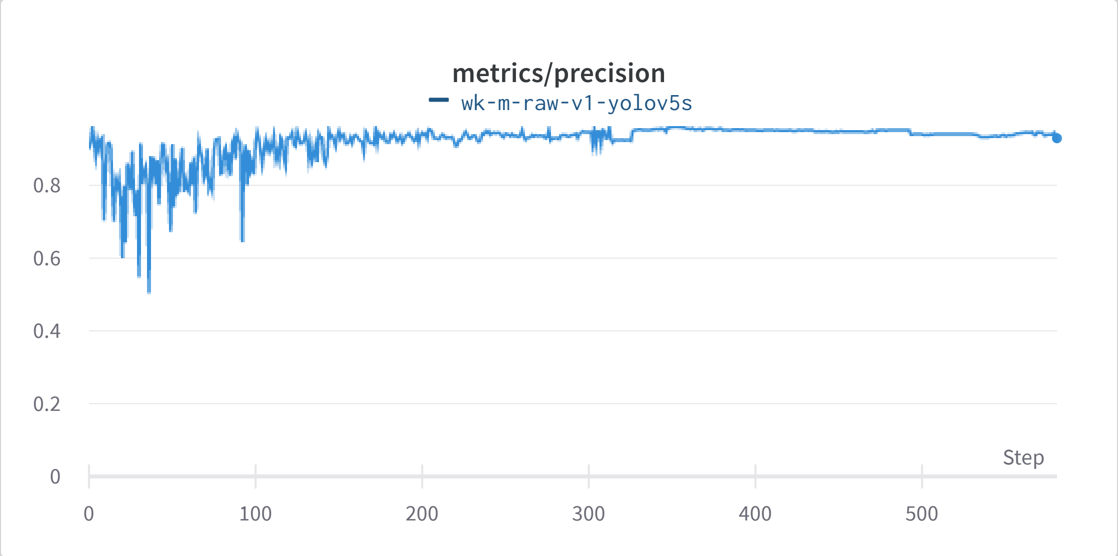
metrics/precision
wk-m-raw-v1-yolov5s
0.8
0.6
0.4
0.2
0
Step
0
100
200
300
400
500

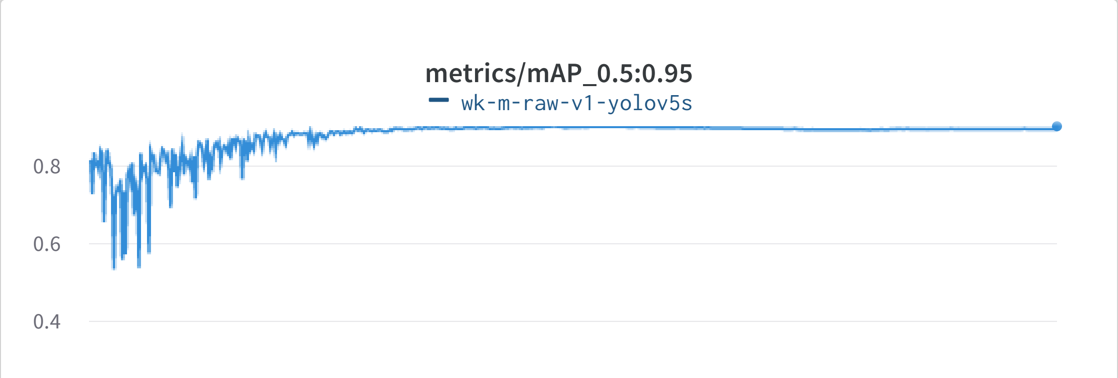
metrics/mAP_0.5:0.95
wk-m-raw-v1-yolov5s
0.8
0.6
0.4

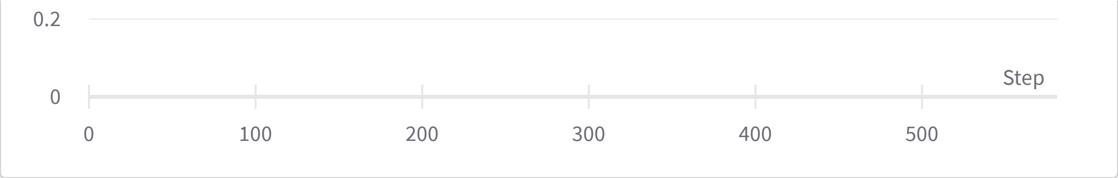
0.2
0
Step
0
100
200
300
400
500

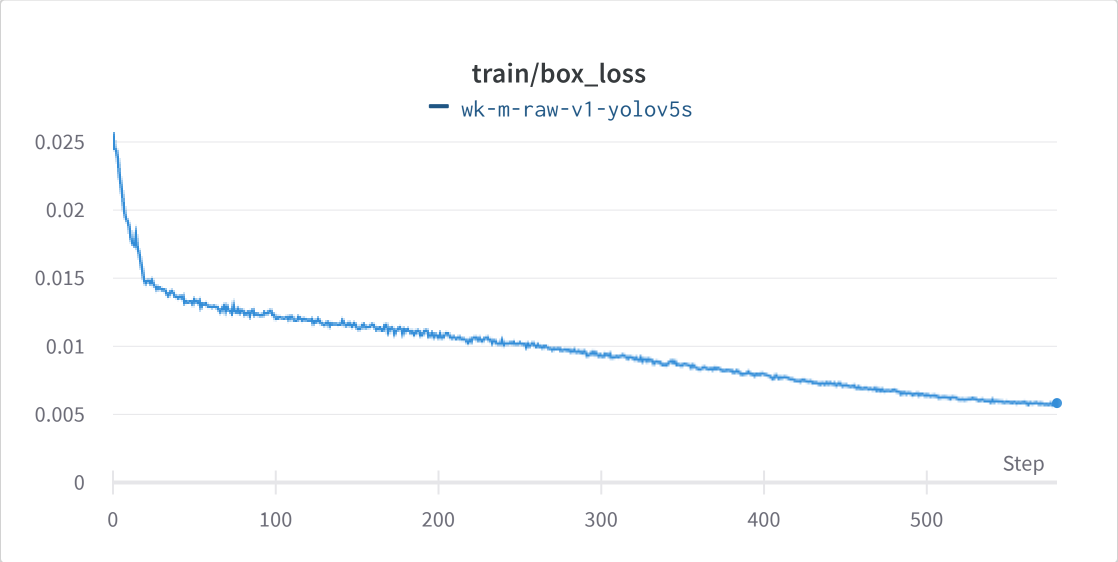
train/box_loss
wk-m-raw-v1-yolov5s
0.025
0.02
0.015
0.01
0.005
0
Step
0
100
200
300
400
500

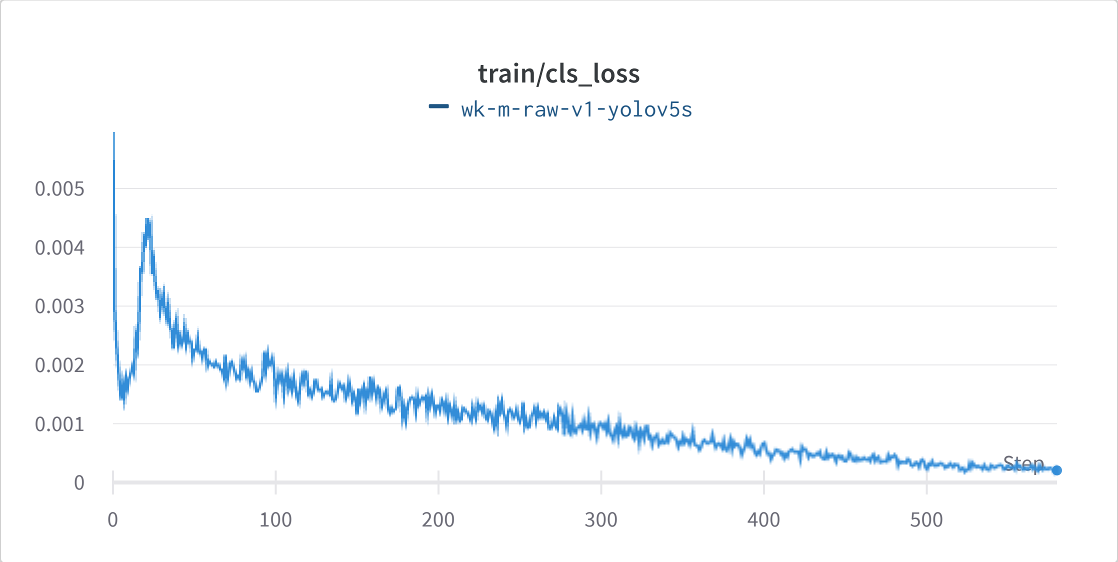
train/cls_loss
wk-m-raw-v1-yolov5s
0.005
0.004
0.003
0.002
0.001
0
Step
0
100
200
300
400
500

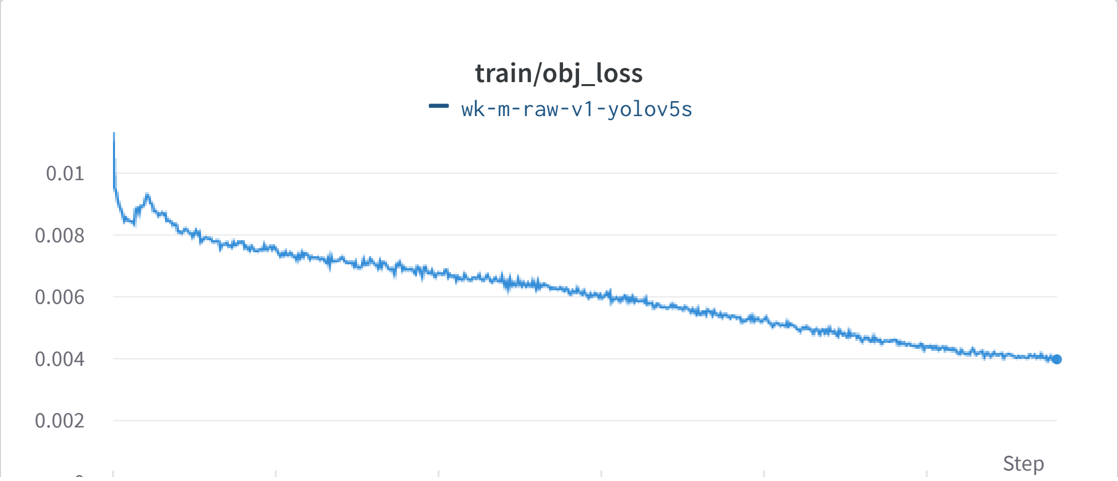
train/obj_loss
wk-m-raw-v1-yolov5s
0.01
0.008
0.006
0.004
0.002
Step

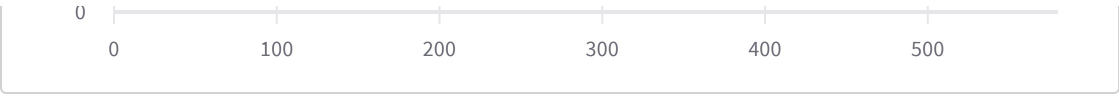
0
0
100
200
300
400
500

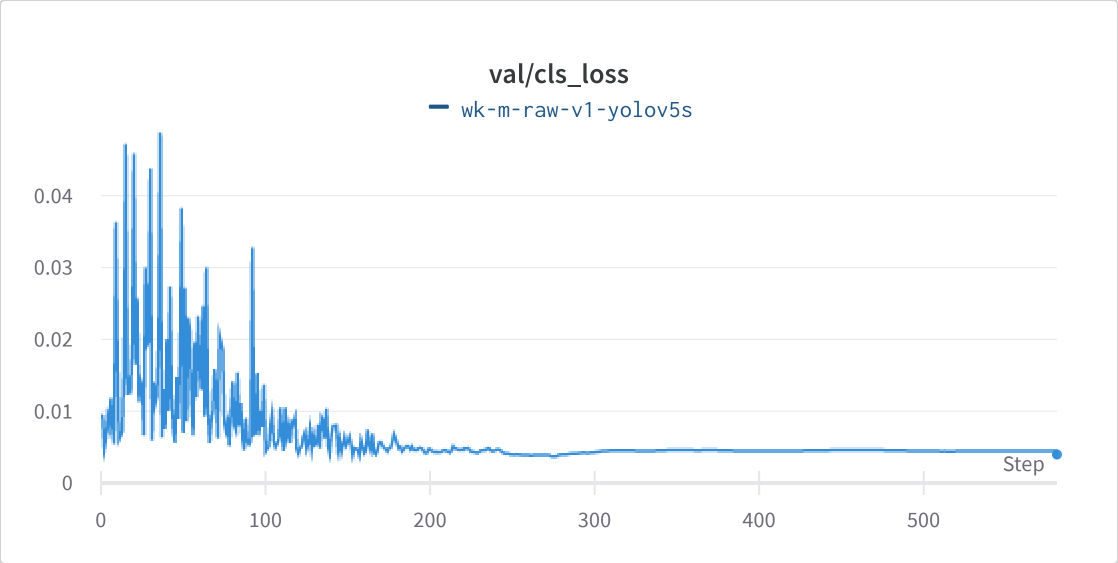
val/cls_loss
wk-m-raw-v1-yolov5s
0.04
0.03
0.02
0.01
0
Step
0
100
200
300
400
500

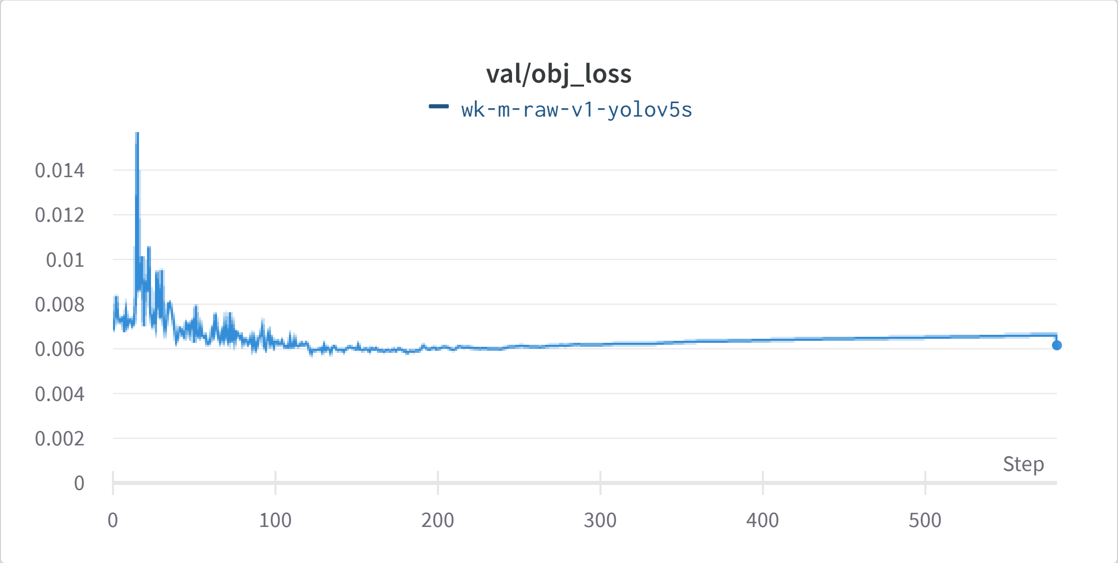
val/obj_loss
wk-m-raw-v1-yolov5s
0.014
0.012
0.01
0.008
0.006
0.004
0.002
0
Step
0
100
200
300
400
500

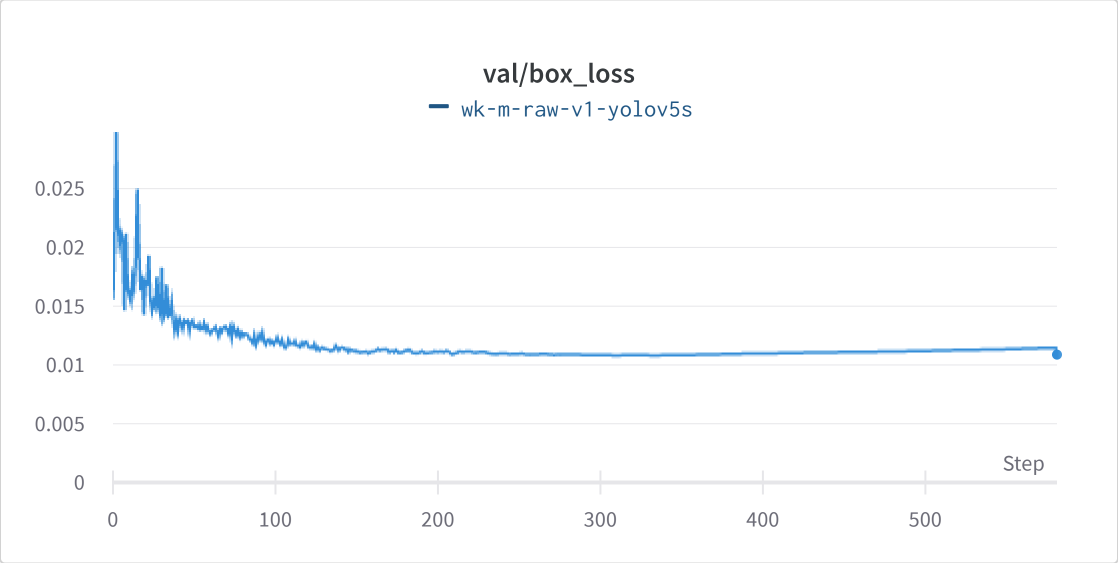
val/box_loss
wk-m-raw-v1-yolov5s
0.025
0.02
0.015
0.01
0.005
0
Step
0
100
200
300
400
500

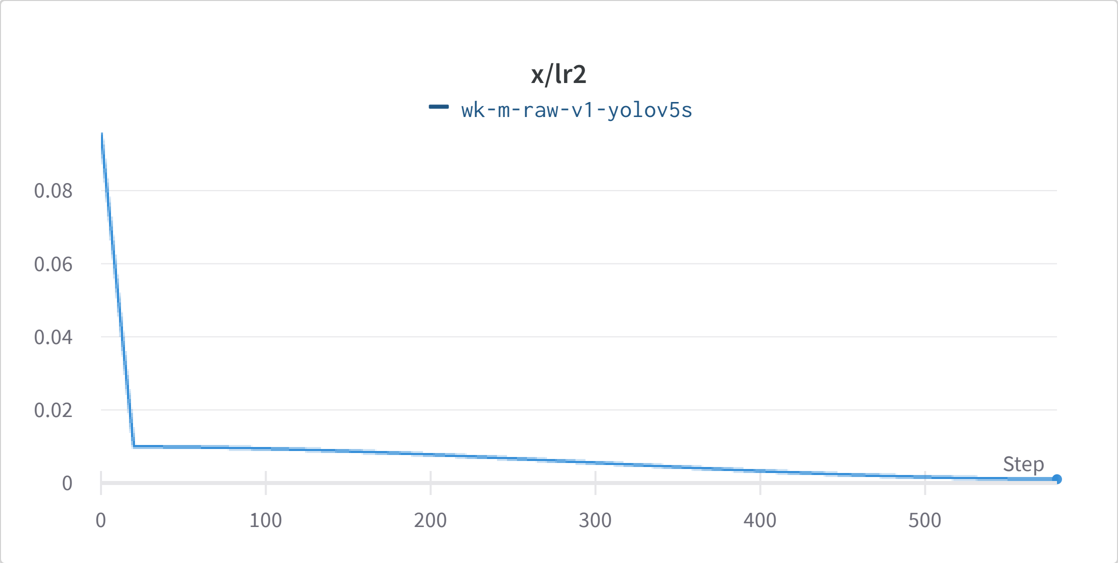
x/lr2
wk-m-raw-v1-yolov5s
0.08
0.06
0.04
0.02
0
Step
0
100
200
300
400
500

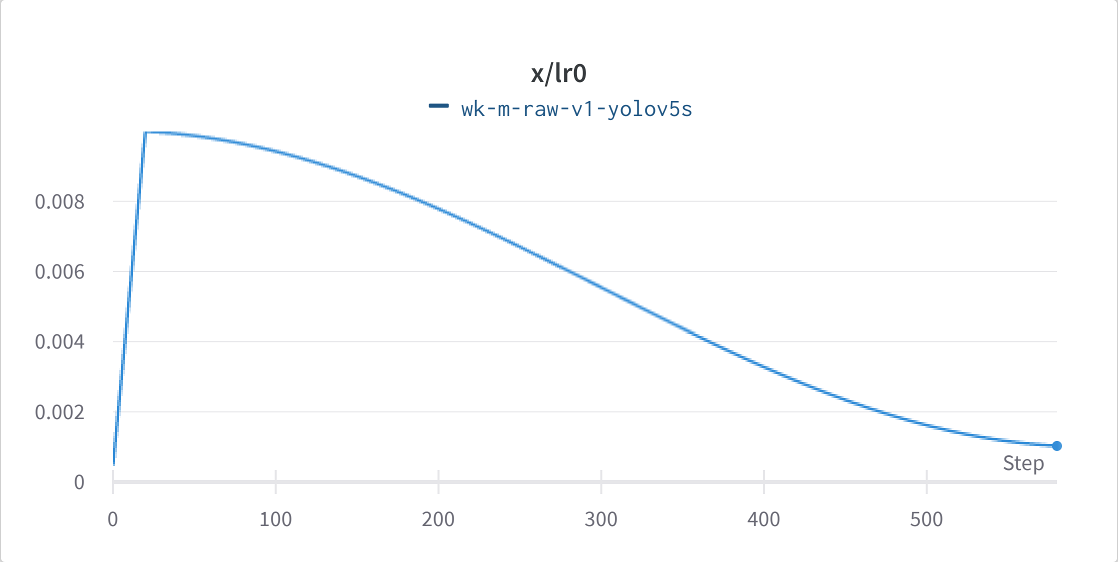
x/lr0
wk-m-raw-v1-yolov5s
0.008
0.006
0.004
0.002
0
Step
0
100
200
300
400
500

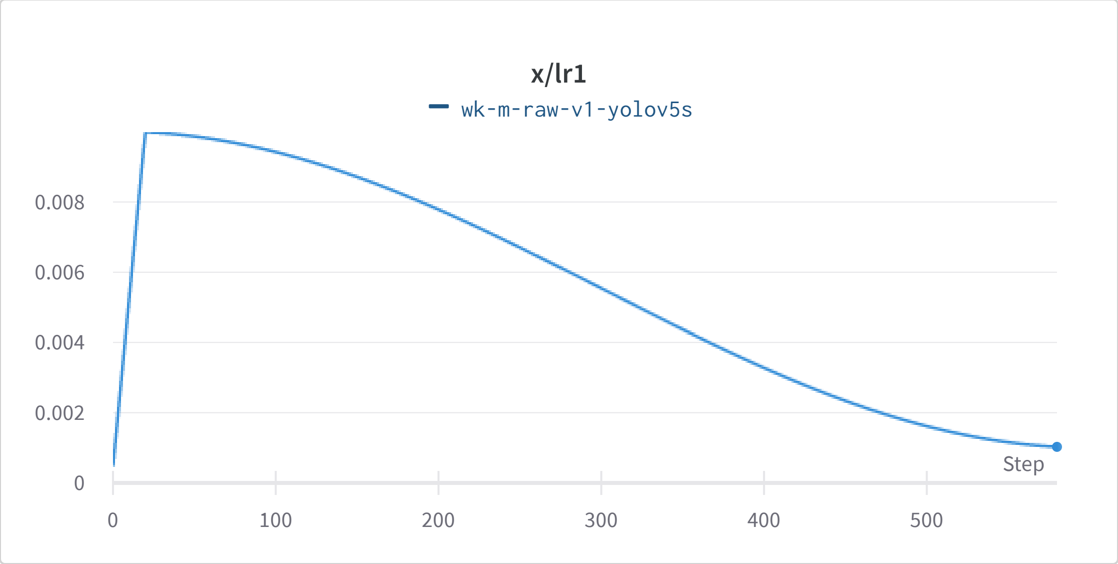
x/lr1
wk-m-raw-v1-yolov5s
0.008
0.006
0.004
0.002
0
Step
0
100
200
300
400
500

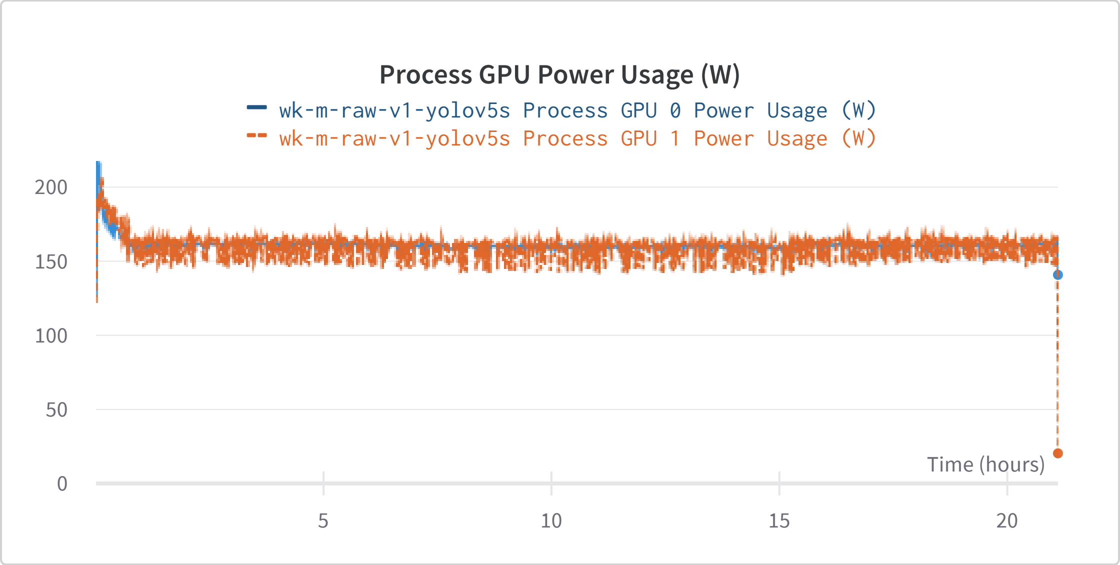

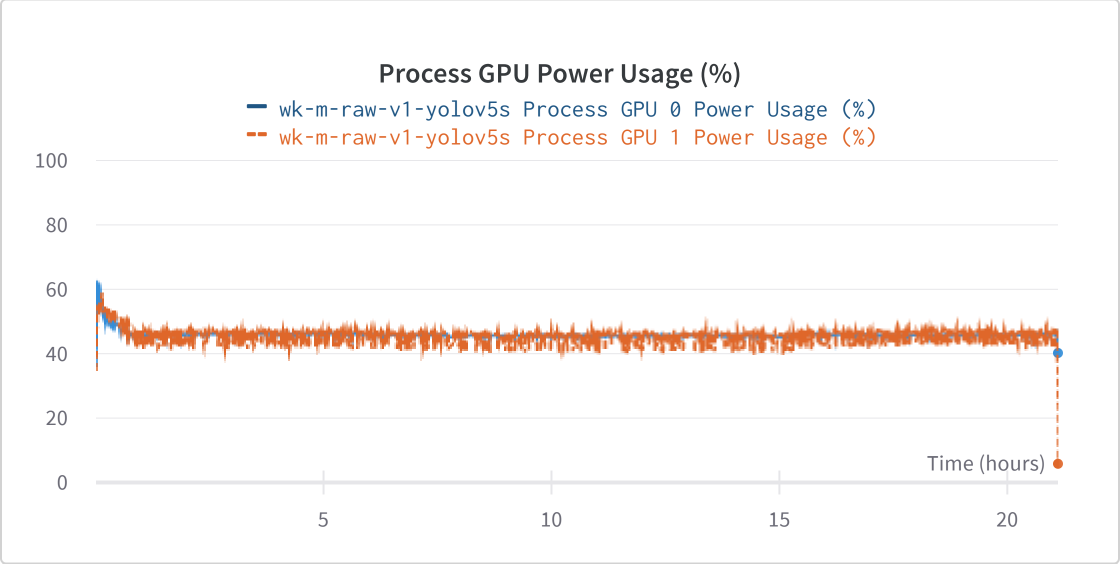

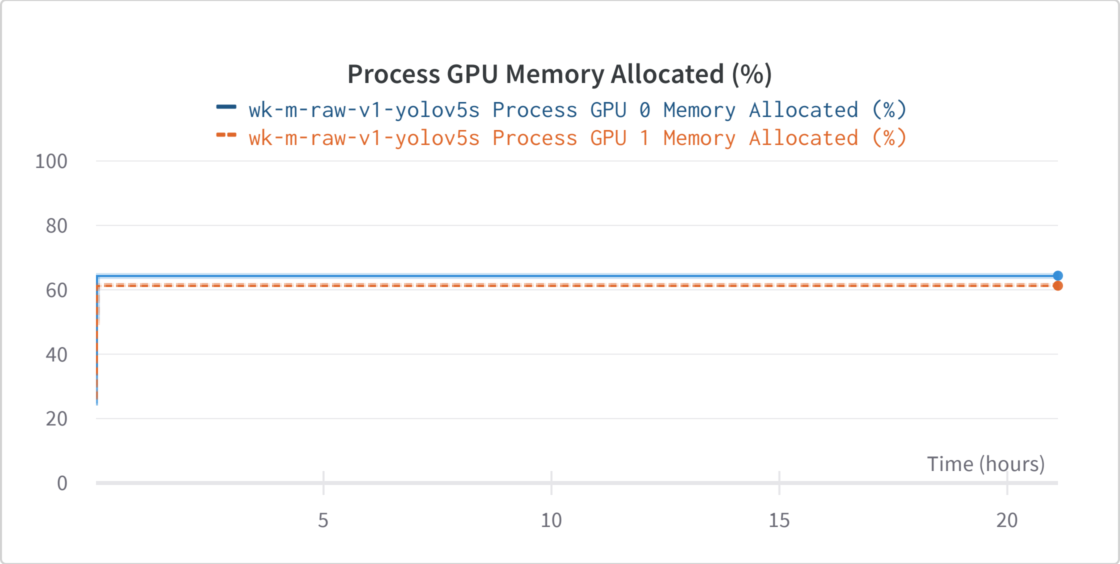

Process GPU Time Spent Accessing Memory (%)

wk-m-raw-v1-yolov5s Process GPU 0 Time Spent Accessing Memory (%)

wk-m-raw-v1-yolov5s Process GPU 1 Time Spent Accessing Memory (%)

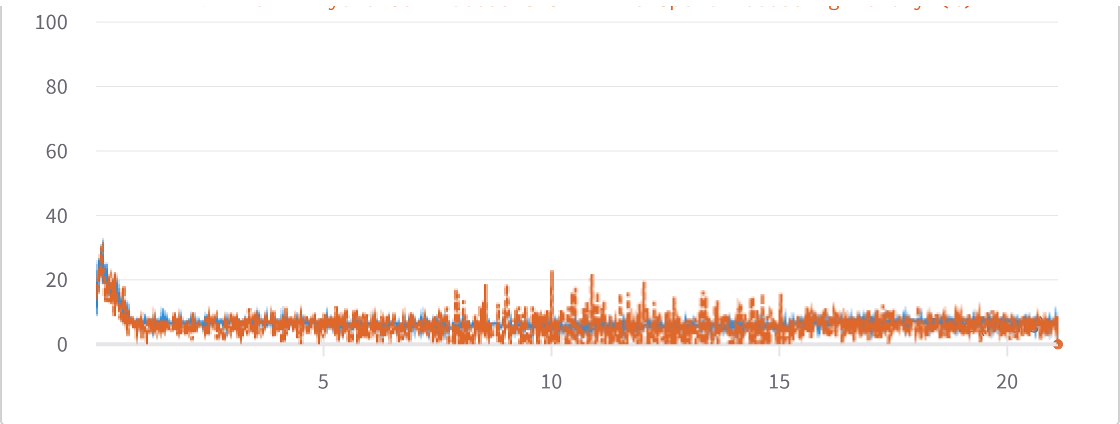

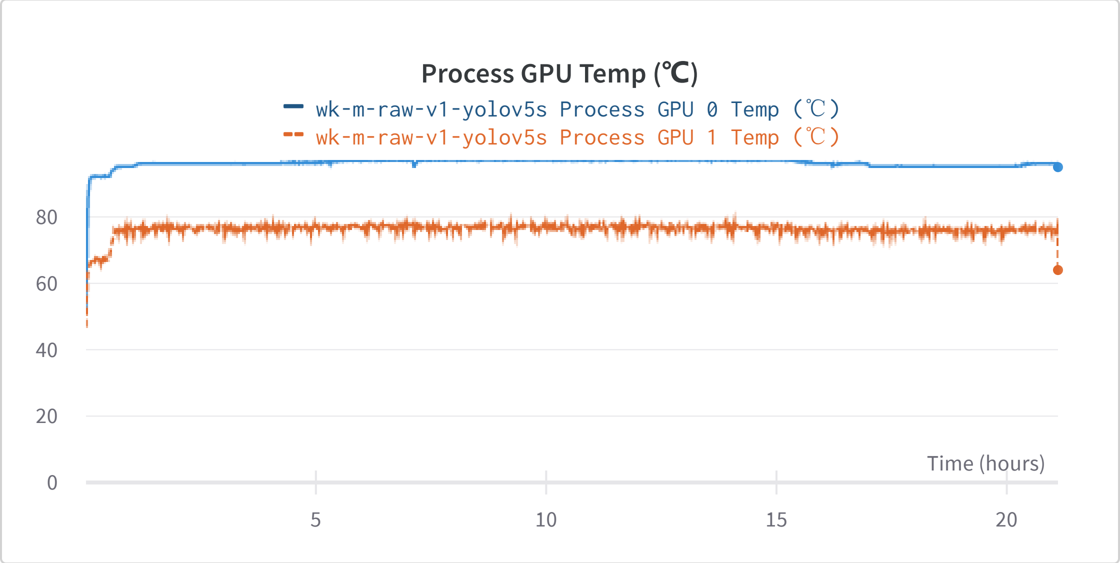

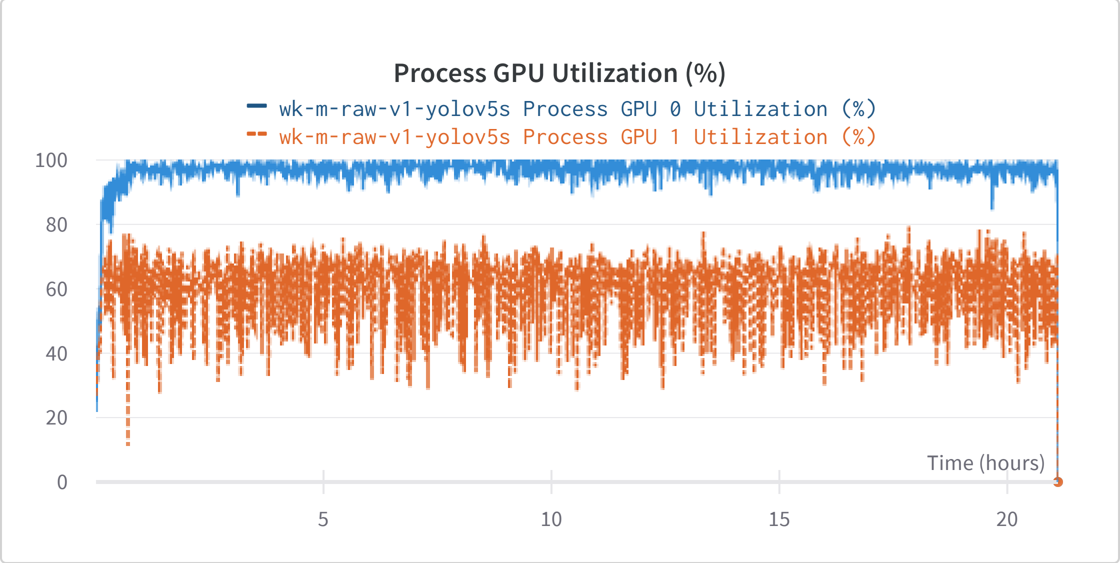

Created with ❤️ on Weights & Biases.

https://wandb.ai/zyt/YOLOv5/reports/Untitled-Report--Vmlldzo0NDcxMjE0
